# Mortality-to-Incidence Ratio for Nasopharyngeal Carcinoma Is Associated with Health Expenditure

**DOI:** 10.3390/healthcare10091615

**Published:** 2022-08-25

**Authors:** Chen Dong, Jing-Tong Fu, Han-Ru Wu, Yu-Chi Chao, Ying-Ching Chen, Wen-Wei Sung, Wen-Jung Chen, Chih-Jung Chen

**Affiliations:** 1 School of Medicine, Chung Shan Medical University, Taichung 40201, Taiwan; 2 Department of Pathology and Laboratory Medicine, Taichung Veterans General Hospital, Taichung 40201, Taiwan; 3 Department of Urology, Chung Shan Medical University Hospital, Taichung 40201, Taiwan; 4 Institute of Medicine, Chung Shan Medical University, Taichung 40201, Taiwan; 5 Department of Post-Baccalaureate Medicine, College of Medicine, National Chung Hsing University, Taichung 40227, Taiwan

**Keywords:** nasopharyngeal carcinoma, mortality, incidence, mortality-to-incidence ratio, expenditure, human development index

## Abstract

Geographic and gender-specific disparity can be observed in nasopharyngeal carcinoma (NPC). While screening and more effective therapies, such as induction chemotherapy, could improve survival rates, they are costly. This study aims to explore the correlation between healthcare expenditure and the mortality-to-incidence ratio (MIR) in NPC. Data were obtained from the World Health Organization and the Global Cancer Observatory. The correlation was evaluated by Spearman’s rank correlation coefficient. Most new cases and deaths occur in Asia, and more males are affected than females. Our study shows that countries with higher MIRs have lower levels of health expenditure regardless of the NPC’s gender-specific incidence. Correspondingly, MIRs are all significantly negatively associated with current health expenditure (CHE) per capita and CHE as a percentage of gross domestic product (CHE/GDP) in both genders. CHE per capita and CHE/GDP have a significant impact on NPC outcomes. Moreover, economic status is a potential major factor in MIR differences between countries.

## 1. Introduction

NPC is an uncommon malignancy arising from the nasopharyngeal epithelium and is distinctly different from other epithelial head and neck tumors. A Global Cancer Observatory (GLOBOCAN) estimate indicated that there were 129,907 new NPC cases in 2018, accounting for just 0.7% of all new cancers [1]; however, the distinct geographic disparity of NPC incidence has raised global concern. NPC is highly prevalent in East and Southeast Asia, where over 70% of new NPC cases are found [2]. In China, the age-standardized incidence rate of NPC in 2018 was 3.0 per 100,000 population, which was about seven times greater than that of white populations (0.4 per 100,000 population) [3]. Interestingly, previous epidemiology studies have found that the NPC rates among second-generation Chinese immigrants are higher than those among local residents, but lower than in the Chinese population in general [4]. Moreover, the farther Chinese immigrants have moved, the lower the incidence of NPC observed. This phenomenon implies that lifestyle, genetic, and environmental factors may influence NPC incidence. These factors include family history of NPC; susceptible genes, including HLA gene (6p21.3), MST1R (3p21.3), and MECOM (3q26); and environmental factors, such as active or passive smoking, preserved food and alcohol consumption, occupational exposure, oral hygiene, and Epstein–Barr virus (EBV) infection, all of which possibly lead to NPC development [4,5]. Among these risk factors, EBV is the leading communal causal agent for the non-keratinizing type of NPC, which accounts for up to 95% of all NPC cases in endemic areas, including China [2]. In addition, the Cantonese ethnicity in southern China has the highest NPC incidence of all ethnicities due to an underlying genetic predisposition [4]. These risk factors might explain the geographic disparity in NPC incidence.

Currently, NPC staging is most commonly determined by MRI, CT, and F-fluorodeoxyglucose (^18^F-FDG) images, since they have the highest sensitivity and accuracy in detecting distant metastasis. The five-year survival rates in stages 1–4 NPC gradually decline, being 92.4%, 85.6%, 74.3%, and 64.9%, respectively [6]. To promote early diagnosis of NPC, the plasma EBV DNA screening test (with a sensitivity of 97.1% and a specificity of 98.6%), a highly effective screening method, is implemented. Compared with historical cohorts, the population which has undergone plasma EBV DNA screening has a higher proportion of stage I–II NPC (71% vs. 20%) and better outcomes (three years free of progression; survival hazard ratio: 0.10, 95% CI 0.05–0.18) than those who have undergone conventional detection methods [6]. Early diagnosis of NPC improves the prognosis of the disease and allows early radiotherapy treatment for stage I NPC. Induction chemotherapy combined with chemoradiotherapy also shows promising results by significantly improving the patients’ survivability [7,8]. Furthermore, intensity-modulated radiotherapy in combination with image-guided radiotherapy is highly recommended to improve the treatment outcomes of NPC (five-year overall survival and disease-free survival rates of 77.9% and 70.5%, respectively) [9]. However, screening tests and more effective therapies require greater health expenditure. Based on a systematic review estimation of EBV DNA polymerase chain reaction, the median cost was USD 18.11 (95% CI = USD 13.35–USD 23.13), and the median cost of transportation to a local laboratory was USD 0.19 (95% CI = USD 0.07–USD 0.89) per person in the United States [10]. In addition, one study outlines the estimate of total cost of induction chemotherapy in China, including gemcitabine plus cisplatin (recurrence-free survival [RFS] USD 23,527.91, recurrence sate USD 29,544.77), cisplatin fluorouracil, and docetaxel (RFS USD 24,461.24, recurrence sate USD 21,021.42). Due to the higher cost of screening tests and more effective therapies, NPC outcomes might depend on both the economy and the government budget.

To test our hypothesis that expenditure on healthcare affects NPC survival, we analyzed the correlation of the mortality-to-incidence ratio (MIR) of NPC with the current health expenditure (CHE) per capita and CHE as a percentage of gross domestic product (CHE/GDP). MIR is a novel parameter to evaluate cancer survival. In this study, we aim to provide a more comprehensive overview of the correlation between NPC mortality outcomes and the economy.

## 2. Materials and Methods

NPC, the International Statistical Classification of Diseases and Related Health Problems 10th Revision (ICD-10 C11), and epidemiological data were obtained from the GLOBOCAN 2018 database (https://gco.iarc.fr/today/ (accessed on 18 September 2020)), which provides publicly accessible data on up-to-date estimates of neoplasm epidemiology in 185 countries. The HDI (human development index) data were obtained from the United Nations Development Program, Human Development Report Office (http://hdr.undp.org/en (accessed on 18 September 2020)). Data on health expenditure, including the CHE per capita and CHE/GDP, were obtained from the World Health Statistics database (https://www.who.int/gho/publications/world_health_statistics/en/ (accessed on 18 September 2020)).

**Figure 1:**
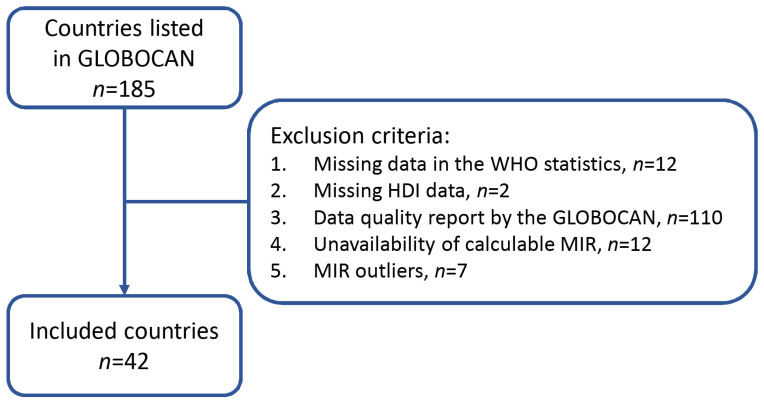
Diagram for data source selection.

MIR was defined as the ratio of the crude rate (CR, per 100,000) of mortality to the CR of incidence, as previously described [11,12,13,14]. The exclusion criteria for country selection were based on missing data in the World Health Organization (WHO) statistics (*n* = 12), no HDI data from the United Nations Development Program, Human Development Report Office (http://hdr.undp.org/en (accessed on 18 September 2020)) (*n* = 2), those with poor data quality reported by GLOBOCAN (*n* = 110, [15]), unavailability of calculable MIR (*n* = 12), and MIR outliers which were below the first quartile (Q1) or above the third quartile (Q3) (*n* = 7). A total of 42 countries was included in the final analysis, as shown in Figure 1. The associations between the MIR, CHE, and CHE/GDP among these 42 countries were analyzed using Spearman’s rank correlation coefficient of bivariate correlations, which was calculated using SPSS statistical software version 15.0 (SPSS, Inc., Chicago, IL, USA). *p* values of <0.05 were considered statistically significant. Scatterplots were generated with SigmaPlot version 12.5 (Chicago, IL, USA).

## 3. Results

### 3.1. Rank, Proportion, Cumulative Risk of New Cases, and Deaths due to NPC by Continent

A total of 127,407 new cases of and 71,208 deaths due to NPC was surveyed in this study. Among various cancers, NPCs are considered to be relatively rare based on the total incidence number. The incidence and mortality case numbers, CR, age-standardized rate (ASR), and MIR are presented in Table 1, which shows a unique geographical distribution pattern. Approximately 85% of new cases and deaths occur in Asia. In addition, the highest cumulative number of new cases and deaths is found in Asia (0.23 and 0.14, respectively), while Latin America and the Caribbean has the lowest cumulative number of new cases (0.04) and Europe, North America, and Latin America and the Caribbean have the lowest cumulative number of deaths (0.02, 0.02, and 0.02, respectively).

### 3.2. CRs of Incidence and Mortality by Country

Table 2 shows the CRs of incidence and mortality by country. The incidence and death CRs in all 42 countries range from 0.13 to 0.08, respectively, in Chile to 10.60, and in Singapore to 5.90. Chile and Ecuador have the lowest incidence CRs (0.13 and 0.15, respectively) and lowest mortality CRs (0.08 and 0.08, respectively) of all countries. Singapore, Malaysia, and Thailand have the highest incidence CRs (10.60, 6.50, and 3.20, respectively) and highest mortality CRs (5.90, 3.70, and 2.00, respectively) of all countries. The incidence CRs of NPC in males are higher than those in females in all considered countries.

### 3.3. MIRs, Relative MIRs, and WHO Ranking by Region and Country

The MIRs of 42 different countries are presented in Table 2. The MIR ranges from 0.27 in Switzerland to 0.72 in Kuwait. The five countries with the highest MIRs are Kuwait, Slovakia, Ukraine, Thailand, and the Philippines (0.72, 0.68, 0.68, 0.63, and 0.63, respectively), all five of which spend no more than USD 1200 per capita on their healthcare system (USD 1169, 1108, 125, 217, and 127, respectively). The CHE per capita and CHE/GDP in these countries are relatively low compared to countries with low MIRs. Switzerland, Croatia, France, Japan, and Ireland have the lowest MIRs (0.27, 0.36, 0.36, 0.37, and 0.37, respectively) and, other than Croatia, higher healthcare expenditure (9818, 852, 4026, 3733, and USD 4757, respectively) than the aforementioned countries. For the correlation of gender difference in MIR, mMIR is significantly correlated to the fMIR (*ρ* = 0.538, *p* < 0.001).

### 3.4. Association of MIRs with CHE per Capita and CHE/GDP by Country

**Figure 2:**
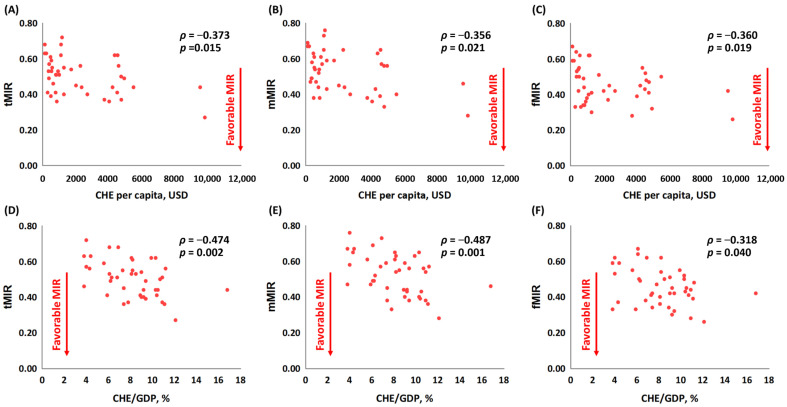
Countries with high current healthcare expenditure (CHE) per capita have low mortality−to−incidence ratios (MIRs) in (**A**) both genders, (**B**) in females, and (**C**) in males. Countries with a high CHE as a percentage of gross domestic product have low MIRs in (**D**) both genders, (**E**) in females, and (**F**) in males.

Figure 2 shows the association between the MIRs and CHE per capita in females, males, and both genders. Lower MIRs, which indicated less mortality crude rates compared to the crude rates of incidence, were the favorable MIRs (Figure 2). The MIRs were all significantly negatively associated with CHE per capita and CHE/GDP (*ρ* = −0.373, *p* = 0.015, Figure 2A; *ρ* = −0.356, *p* = 0.021, Figure 2B; *ρ* = −0.360, *p* = 0.019, Figure 2C; *ρ* = −0.474, *p* = 0.002, Figure 2D; *ρ* = −0.487, *p* = 0.001, Figure 2E; and *ρ* = −0.318, *p* = 0.040, Figure 2F, respectively).

## 4. Discussion

This is the first study to explore the association between MIR, CHE per capita, and CHE/GDP to enhance healthcare planning for NPC programs. Our study showed a negative linear correlation of country-specific MIRs with CHE per capita and CHE/GDP, indicating that countries with higher CHE per capita and CHE/GDP have lower MIRs. Development status may reflect healthcare expenditure in individual countries, which would have an impact on NPC outcomes. Due to the expense of the current screening tests and treatment options, the prognosis of NPCs might be linked to economic status. However, compared with cancers with more established or feasible screening strategies and more therapeutic options, the correlations between MIR and healthcare expenditure on NPC are not as significant as correlations with lung cancer, colorectal cancer, gastric cancer, and lip and oral cancer [16,17,18,19].

Over the past few decades, many epidemiological studies have shown a gradual decline in the incidence and mortality of NPC [20] in most countries. These declining trends may have been the result of a better understanding of the pathogenesis and risk factors of NPC, the development of early detection systems (e.g., EBV DNA screening), and the use of other screening methods to detect distant metastasis with great accuracy (e.g., MRI, CT, and ^18^F-fluorodeoxyglucose-PET/CT) [21,22]. For instance, one-time EBV DNA screening makes an earlier diagnosis of different stages of NPC possible. In one study, one-time EBV DNA screening had 43% stage I, 24% stage II, 32% stage III–IVB, and 1% stage IVC occurrence, in contrast with the 6%, 29%, 54%, and 11% occurrence, respectively, of traditional detection methods in the Asian American population. All the aforementioned factors that might reduce NPC incidence and mortality require additional health expenditure. For example, one-time EBV DNA screening adds an additional USD 63 per 0.00055 quality-adjusted life years (QALY) gain, or, in other words, USD 113,341 per QALY gain. EBV DNA screening increases men’s QALY by 2.0 when compared to usual care without screening [23]. However, in comparison with other detection strategies, including positron emission/computerized tomography, computerized tomography and head and neck magnetic resonance imaging, chest X-ray, abdominal ultrasonography, and bone scan, the EBV DNA screening test remains more cost-effective [24]. Moreover, in patients with primary stage T1–4 NPC, the cost-effectiveness for annual and biannual surveillance MRI lies in the range of 0.022–0.088 QALYs when compared with non-MRI follow-up. Hence, the incremental cost effectiveness ratios (ICERs) for annual and biannual MRI detection method are in the range of USD 169,772–USD 403,857 [25]. Even though the cost-effectiveness of early detection systems and other screening methods for distant metastasis varies in different populations, age groups [10], and genders, an individual country’s economy would still have a huge impact on local healthcare policy and NPC outcomes. These findings highlight the importance of the economy in clinical outcomes, since richer countries can afford earlier screening methods, which is consistent with the negative association between MIRs and healthcare expenditure shown by our results.

Treatment also plays an important role in NPC prognosis. The most recent NPC therapies, including intensity-modulated radiation therapy (IMRT), chemotherapy, and immunotherapy, highly improve NPC outcomes [26]. IMRT is currently the standard therapy for any stage of NPC and utilizes complex modulated radiation to have sharper radiation dose gradients than conventional two-dimensional radiotherapy (2D-RT). It also has a loco-regional relapse-free survival rate of 95.6, which is better than 2D-RT [27,28]. Despite the remarkable performance of IMRT in NPC outcomes, this study shows IMRT is more expensive than the traditional radiation treatment. For patients with early stage tumor, IMRT would cost USD 95,047, while surgery alone and traditional radiation treatment would only cost USD 18,140 and USD 32,296, respectively. For later-stage tumor treatment, the cost of chemotherapy would be USD 98,624, compared to the USD 29,656 for patients who did not receive it. If concomitant chemotherapy for later-stage tumor is excluded, IMRT would cost USD 67,576, and non-IMRT treatment would cost USD 24,955. [29]. Having concurrent chemotherapy and IMRT in addition to radiotherapy highly improves the NPC locoregional control rate, as supported by previous research, but additional treatment plans would increase healthcare spending [7,30]. These results further support the significant negative association between MIRs and healthcare expenditure in our results.

Several studies have reported lower NPC incidence rate, mortality rate, and MIR in women than in men, which indicates a better NPC prognosis in women than in men [20,31]. Previously, the disparity in NPC MIR was assumed to be due to differences in lifestyle behavior, health-seeking behavior, and biological traits such as sex hormones. However, another study found that postmenopausal women have a similar NPC incidence and mortality to men, which further supports the hypothesis that intrinsic biological hormones such as estrogen play an important role in the better prognosis seen in female than in male patients [31]. This phenomenon is consistent with our results only to a certain degree. However, we suggest factors besides biological traits, such as disparity in healthcare expenditure for females and males, should be considered in future research to enable a more holistic view of this gender-specific trend.

This study provides an overview of the relationship between MIR, CHE per capita, and CHE/GDP in 42 countries. The data are extracted from the GLOBOCAN database, which provides validified and high-quality data. However, our study has several limitations. First of all, some of the data might be missing in resource-limited countries because they may not have adequate financial strength in their healthcare system to accurately keep track of all their cancer statistics, possibly resulting in the distortion of incidence and mortality rates. Furthermore, health expenditure in countries could be correlated with overall societal wealth. Secondly, our study only includes countries whose data are recorded by the WHO, which may cause some selection bias. Thirdly, MIR might not be a sufficient target to replace cancer survival rate or long-term prognostic data [32]; however, we believe MIR is an appropriate tool to identify cancer control and examine worldwide cancer-screening and treatment programs. Fourthly, the data obtained from GLOBOCAN were estimates in certain countries, which might have some impact on the study results; we have tried to minimize the effect by excluding countries with missing data. Fifthly, some important risk factors for NPC, such as gender, tobacco control, and diet behavior changes, are not recorded or analyzed. Nevertheless, our study plays a consequential role in countries where NPC survival data are unavailable and have important implications for how their healthcare policy making could improve their NPC incidence and mortality. To our knowledge, this study is the first to use MIR to systematically demonstrate the association between NPC prognosis and healthcare expenditure and thus enables better planning for future NPC programs.

## 5. Conclusions

MIR is significantly negatively associated with CHE per capita and CHE/GDP in both genders. CHE per capita and CHE/GDP have a significant impact on NPC outcomes, indicating that economic status is a potential major factor in the MIR differences between countries. More effective screening tests and treatments are available in countries with higher CHE per capita and CHE/GDP to reduce NPC incidence, mortality, and MIR in the long run and to improve survival rates. Therefore, countries with high NPC prevalence may need a certain level of economic capacity to reduce NPC mortality further.

## Figures and Tables

**Table 1:** Summary of the incidence, mortality, and mortality-to-incidence ratio in nasopharyngeal cancer of selected regions.

	New Cases				Deaths				Mortality-to-Incidence Ratio
Region	Number	Crude Rate	Age-Standardized Rate	Cum. Risk	Number	Crude Rate	Age-Standardized Rate	Cum. Risk	
All regions	127,407	1.70	1.50	0.16	71,208	0.94	0.83	0.10	0.55
Africa	9418	0.73	1.00	0.11	5564	0.43	0.67	0.08	0.59
Asia	108,061	2.40	2.10	0.23	60,926	1.30	1.20	0.14	0.54
Europe	4857	0.67	0.43	0.05	2397	0.33	0.19	0.02	0.49
Latin America and the Caribbean	2502	0.39	0.35	0.04	1167	0.18	0.16	0.02	0.46
North America	2332	0.65	0.45	0.05	1025	0.29	0.17	0.02	0.45
Oceania	237	0.58	0.47	0.05	129	0.32	0.24	0.03	0.55

**Table 2:** Summary of current health expenditure, incidence, mortality, and mortality-to-incidence ratio in nasopharyngeal cancer.

	Current Health Expenditure		Incidence, Crude Rate			Mortality, Crude Rate			Mortality-to-Incidence Ratio		
Country	Per Capita	% of GDP	Total	Female	Male	Total	Female	Male	Total	Female	Male
Argentina	998	6.8	0.35	0.24	0.47	0.18	0.09	0.27	0.51	0.38	0.57
Australia	4934	9.4	0.65	0.34	0.96	0.32	0.11	0.54	0.49	0.32	0.56
Austria	4536	10.3	0.42	0.23	0.62	0.26	0.12	0.40	0.62	0.52	0.65
Belarus	352	6.1	0.38	0.22	0.55	0.20	0.14	0.27	0.53	0.64	0.49
Belgium	4228	10.5	0.64	0.29	1.00	0.28	0.13	0.43	0.44	0.45	0.43
Brazil	780	8.9	0.49	0.29	0.70	0.20	0.10	0.31	0.41	0.34	0.44
Bulgaria	572	8.2	1.10	0.60	1.60	0.61	0.37	0.86	0.55	0.62	0.54
Canada	4508	10.4	0.76	0.44	1.10	0.31	0.19	0.43	0.41	0.43	0.39
Chile	1102	8.1	0.13	0.10	0.17	0.08	0.04	0.11	0.62	0.40	0.65
Colombia	374	6.2	0.37	0.18	0.57	0.18	0.09	0.28	0.49	0.50	0.49
Costa Rica	929	8.1	0.73	0.45	1.00	0.39	0.16	0.61	0.53	0.36	0.61
Croatia	852	7.4	0.74	0.29	1.20	0.27	0.10	0.45	0.36	0.34	0.38
Cuba	826	10.9	1.80	0.77	2.80	0.91	0.34	1.50	0.51	0.44	0.54
Czechia	1284	7.3	0.53	0.27	0.79	0.29	0.11	0.47	0.55	0.41	0.59
Denmark	5497	10.3	0.41	0.28	0.53	0.18	0.14	0.21	0.44	0.50	0.40
Ecuador	530	8.5	0.15	0.10	0.20	0.08	0.05	0.11	0.53	0.50	0.55
France	4026	11.1	0.75	0.28	1.20	0.27	0.11	0.43	0.36	0.39	0.36
Germany	4592	11.2	0.52	0.27	0.77	0.29	0.13	0.44	0.56	0.48	0.57
Ireland	4757	7.8	0.46	0.17	0.76	0.17	0.08	0.25	0.37	0.47	0.33
Italy	2700	9.0	1.00	0.48	1.50	0.40	0.20	0.60	0.40	0.42	0.40
Jamaica	294	5.9	1.10	0.63	1.50	0.45	0.21	0.70	0.41	0.33	0.47
Japan	3733	10.9	0.73	0.40	1.10	0.27	0.11	0.42	0.37	0.28	0.38
Kuwait	1169	4.0	0.69	0.45	0.87	0.50	0.28	0.66	0.72	0.62	0.76
Malaysia	386	4.0	6.50	3.20	9.70	3.70	1.70	5.60	0.57	0.53	0.58
Netherlands	4746	10.7	0.46	0.29	0.63	0.23	0.12	0.35	0.50	0.41	0.56
Oman	636	3.8	0.46	0.18	0.59	0.21	0.06	0.28	0.46	0.33	0.47
Philippines	127	4.4	2.70	1.70	3.60	1.70	1.00	2.40	0.63	0.59	0.67
Poland	797	6.3	0.70	0.45	0.97	0.36	0.22	0.50	0.51	0.49	0.52
Portugal	1722	9.0	1.40	0.61	2.20	0.76	0.31	1.30	0.54	0.51	0.59
Russian Federation	524	5.6	0.41	0.20	0.64	0.24	0.11	0.39	0.59	0.55	0.61
Serbia	491	9.4	1.30	0.55	2.10	0.51	0.23	0.80	0.39	0.42	0.38
Singapore	2280	4.3	10.60	6.20	15.00	5.90	2.30	9.70	0.56	0.37	0.65
Slovakia	1108	6.9	0.71	0.29	1.10	0.48	0.18	0.80	0.68	0.62	0.73
South Africa	471	8.2	0.33	0.24	0.43	0.20	0.13	0.27	0.61	0.54	0.63
South Korea	2013	7.4	0.76	0.33	1.20	0.34	0.14	0.54	0.45	0.42	0.45
Spain	2354	9.2	0.81	0.42	1.20	0.36	0.19	0.53	0.44	0.45	0.44
Switzerland	9818	12.1	0.63	0.38	0.87	0.17	0.10	0.24	0.27	0.26	0.28
Thailand	217	3.8	3.20	1.70	4.60	2.00	1.00	3.10	0.63	0.59	0.67
Ukraine	125	6.1	0.57	0.33	0.84	0.39	0.22	0.58	0.68	0.67	0.69
United Kingdom	4356	9.9	0.39	0.20	0.59	0.24	0.11	0.37	0.62	0.55	0.63
United States of America	9536	16.8	0.64	0.36	0.92	0.28	0.15	0.42	0.44	0.42	0.46
Uruguay	1281	9.2	0.68	0.40	0.97	0.27	0.12	0.42	0.40	0.30	0.43

## Data Availability

The datasets used and/or analyzed during the current study are publicly available in the Global Cancer Observatory (GLOBOCAN) database (https://gco.iarc.fr/today/, accessed on 18 September 2020), United Nations Development Program/Human Development Report Office (http://hdr.undp.org/en, accessed on 18 September 2020) and World Health Statistics database (https://www.who.int/gho/publications/world_health_statistics/en/, accessed on 18 September 2020).
